# A Qualitative Analysis of the Culture of Antibiotic Use for Upper Respiratory Tract Infections Among Patients in Northwest Russia

**DOI:** 10.3389/fphar.2022.800695

**Published:** 2022-01-31

**Authors:** Lourdes Cantarero-Arevalo, Lotte S. Nørgaard, Sofia K. Sporrong, Ramune Jacobsen, Anna Birna Almarsdóttir, Johanne M. Hansen, Dmitry Titkov, Svetlana Rachina, Ekaterina Panfilova, Viktoria Merkulova, Olga Eseva, Nadezhda Riabkova, Susanne Kaae

**Affiliations:** ^1^ WHO Collaborating Centre for Research and Training in the Patient Perspective on Medicine Use, Department of Pharmacy, University of Copenhagen, Copenhagen, Denmark; ^2^ Department of Pharmacy, Uppsala University, Uppsala, Sweden; ^3^ Finnish Institute for Health and Welfare (THL), Helsinki, Finland; ^4^ Sechenov First Moscow State Medical University, Moscow, Russia; ^5^ St. Petersburg City Centre for Medical Prevention, St. Petersburg, Russia; ^6^ Arkhangelsk Regional Centre for Public Health and Medical Prevention, Arkhangelsk, Russia; ^7^ Pskov Regional Public Health Centre, Pskov, Russia; ^8^ Petrozavodsk State University, Institute of Medicine, Petrozavodsk, Russia; ^9^ Republican Hospital V. A. Baranova, Petrozavodsk, Russia

**Keywords:** antimicrobial resistance, antibiotics, urtis, patient perspective, qualitative, attitudes, Russia

## Abstract

**Introduction:** Due to the globally persistent threat of Antimicrobial Resistance (AMR), the purpose of this study was to gain an in-depth understanding of the antibiotic (AB) practices, knowledge and attitudes among patients residing in five regions in the northwest part of Russia. Given the high prevalence, this study focused on ABs for Upper Respiratory Tract Infections (URTI).

**Methods:** The qualitative, semi-structured interviews followed a guide organized by major themes such as common symptoms, consultations with doctors and external influences in decision-making. Patient participants were recruited *via* convenience sampling. Fifty-five interviews were conducted among patients using ABs for URTIs purchased with or without prescription. Data was analyzed using a direct content analysis and validation rounds were conducted between interviewers and data analyzers.

**Results:** Self-medication with ABs seemed a common practice across all five Russian regions; in some cases, patients tried to persuade pharmacists into selling them ABs without prescription. Factors, such as time spent going to the doctor, need of a sick leave or self-persuasion, influenced the decisions of whether or not to seek the doctor for symptoms of URTIs. Knowledge of ABs and AMR was generally low; however, some patients with seemingly good knowledge practiced self-medication from time to time. Family members and friends were often involved in decisions about how to handle symptoms of URTIs, especially among those patients using ABs without prescription. Few patients had noticed ABs awareness campaigns, and very few reported having learned something important from them.

**Conclusion:** Despite enforced regulation of AB use in Russia, self-medication still exists. Knowledge is not always linked to appropriate use of AB, and the few campaigns conducted were not always noticed.

## Introduction

Despite the increasing number of governments around the world devoting attention and resources to revert and reduce Antimicrobial Resistance (AMR) (1), AMR continues to threaten the effective antibiotic (AB) treatment of an increasing range of infections caused by bacteria, parasites and fungi (Ferri et al., 2017). A study conducted in the WHO European Region showed that inappropriate ABs use (either over- or underuse) was high in many WHO-Region countries, including Eastern European and Central Asia countries, including Russia (Versporten et al., 2014). Many factors may contribute to this culture of inappropriate ABs use such as prescribing habits, sometimes determined by patients’ demands for ABs, and self-treatment, possibly due to the easy availability of ABs without a prescription (Ferri et al., 2017).

In order to strengthen appropriate ABs use and to prevent further AMR, a deeper and more nuanced understanding of patients’ and health care professionals’ (HCP) knowledge, attitudes and actual behaviors related to AB is needed. Studies of this sort have been carried out during the recent years in a number of WHO European Region countries. The combination of multiple aspects involved in patients’ and HCPs’ AB decision-making imply that these processes are more complex than previously reported (Kaae et al., 2017; Arianit et al., 2019; Kaae et al., 2020). Beyond common patterns identified across countries, those complex processes show the relevance of understanding in depth the subnational or local cultures linked to ABs use. Russia is one of the countries currently devoting a lot of attention to tackle AMR (National Strategy for Com, 2030), therefore a study shedding light on the regional patterns of AB use could be of value to inform AMR containment policies, and respective action plans.

Russia, along with Brazil, India, China, and South Africa, accounted for 76% of the overall increase in the global consumption of antibiotics (Van Boeckel et al., 2014; NetworkOfA 2021) and it belongs to the group of countries with moderate consumption of AB (National Strategy for Com, 2030; NetworkOfA 2021). Over-the-counter access to antimicrobials, including antituberculosis drugs, was at some moment common, and self-medication became increasingly popular in the Russian society (Balabanova et al., 2004). The Russian legislation states that ABs can only be dispensed against prescriptions; however, this prerequisite is not necessarily enforced. This leads to arbitrary attitudes toward ABs among health professionals, especially pharmacists (Belkina et al., 2017). A significant increase in the prevalence of multiresistant bacterial pathogens has been observed among both inpatients and outpatients in Russia, including northern regions of Russia (Kalinogorskaya et al., 2015; Palagin et al., 2020; Kuzmenkov et al., 2021). In order to combat this growing problem, the national strategy to prevent the spread of AMR and plan for its implementation were approved by the Russian government in 2017 and 2020, respectively. A number of national guidelines targeting appropriate AB use are available, and some interventions aimed at reducing AMR in Russia have demonstrated a beneficial effect on the practice of AB use. However, more initiatives are needed (BodyaevaEVR et al., 2011; GusarovVGN et al., 2015).

Therefore a collaboration between the Finnish Institute for Health and Welfare (THL) and the WHO Collaborating Center for Research and Training in the Patient Perspective on Medicine use (WHO CC MedUse), University of Copenhagen, under the programme of the “Nordic-Russian Cooperation on Antimicrobial Resistance (AMR) Containment” was established. This collaboration was part of the Nordic Council of Ministers’ Thematic Programme for Health Promotion and Prevention. The aim of the collaboration was to study the culture of use of ABs in Northwest Russia to inform future AMR programmes. The partner-regions from the Russian side in the Nordic-Russian Cooperation on AMR Containment were Arkhangelsk Region, Karelia Republic, Murmansk Region, Pskov Region and St. Petersburg City, and these five regions were included in the study.

The specific aim of this study was to investigate AB practices, knowledge and attitudes of patients in the five regions in Northwest Russia in order to get a deeper understanding of the reasons underlying the use of AB.

## Methods

The project investigated the practices, knowledge, and attitudes towards AB use for upper respiratory tract infections (URTIs) of patients and healthcare professionals, as the use of AB is assumed to be influenced by both parties. The particular disease area of symptoms related to URTI was chosen in order to make results comparable, also because this area constitutes a large proportion of inappropriately used ABs. The use of interviews was chosen as it has previously proven to be an appropriate method to study practices, knowledge, and attitudes related to use of AB, as this method can reveal details of the situations in which AB is prescribed, purchased and taken (Kaae et al., 2016). The results of interviews with healthcare professionals (pharmacists and doctors) will be reported in another paper.

### Training of National Research Teams

A training seminar organized and delivered by the WHO CC MedUse, took place in October 2019 in Pskov, Russia. The purpose of this seminar was to prepare representatives from the five regions (Arkhangelsk, Karelia, Murmansk, Pskov and St. Petersburg) to conduct interviews, manage qualitative data and support the analysis of the data gathered. The seminar covered an introduction to qualitative research and practical exercises. It also included teaching on how to conduct a good interview and different roles of an interviewer. Practicalities of the process of interviewing such as recruitment, recording and transcription were covered along with ethical considerations. The training seminar ended with an explication of the tasks ahead including deadlines.

### Study Population and Recruitment

Following the advices and indications provided by the WHO CC MedUse researchers, each region decided how to recruit patients for the interviews. In most cases, professional networks and the ‘snowball’ recruitment strategy were applied. Second level networks (friends of friends) were in some cases also recruited for patient interviews. Each region conducted individual interviews with an average of 10 adult patients (over 18 years old) who had used one or more of the following ABs: amoxicillin-clavulanic acid, azithromycin, levofloxacin or ceftriaxone to treat symptoms for URTIs during the last 3 months. The pharmaceuticals were chosen as these specific ABs are central with regard to rational use of ABs for URTI. Both patients who had received ABs through a prescription and without a prescription were included in the study.

### Data Collection Methods

The regional teams used two different versions of the interview-guide, one for patients with a prescription and one for patients without a prescription. The aim of the interviews was to provide data regarding knowledge, behaviours and attitudes of patients in relation to the following items:

• The process of diagnosis/purchase
• Why a specific AB was selected
• Where and how an AB was purchased
• Satisfaction with the AB purchase process
• Knowledge on ABs, and
• Attitudes towards ABs

The precise themes and sub-themes varied slightly between the two groups of patients. The interview-guides were divided in two overall parts:

a) a detailed description of the last time the interviewee got an AB prescription or the last time they purchased the AB in the pharmacy.
b) a description/reflection about how the interviewee would compare the last time the AB was prescribed/purchased, with the way it is usually prescribed/purchased.

The reason for this division was that nuanced results on AB behavior and attitudes could more easily be derived through narratives on how the interviewees react in specific situations concerning ABs. However, to explore if this incident was typical of common practices, the resemblance to other similar situations was taken into account. Most interviews were audio-recorded. The interviews usually took place in a café or in the patient’s home according to the preferences of the participants. Eleven local researchers–either doctors or pharmacists-conducted the interviews. Two persons conducted the interviews in Arkhangelsk, Karelia, Murmansk and Pskov and three in Skt. Petersburg. The interviewers filled out the ‘Scheme on participants and sampling criteria’, hence reporting which specific sampling criteria and recruitment technique was used. They specified gender, age, and educational level of participants, however, all personal data about interviewees was completely anonymized.

### Data Management and Analysis

All interviews were conducted in Russian and were transcribed by the interviewers. Pauses or expressions of sounds were not transcribed. The transcriptions were done in an anonymized way; i.e. deleting names and other factors that could be identified. The transcripts were sent to THL, who translated them into English and then sent it to WHO CC MedUse for analysis. Researchers from the WHO CC Meduse validated the collected data. First, the transcriptions and their translations from Russian into English underwent scrutiny by a bilingual expert and precisions were made. Then, the way the first interviews had been conducted was assessed with regard to the interview technique and some recommendations were sent back to the interviewers through the THL to improve the quality of the interviews. For example, there was a need to pay more attention to specific issues and ask all questions included in the interview-guides and/or to make sure to ask for specific episodes.

The data collected were then analyzed applying a directed content analysis (i.e., in a deductive way) (Hsieh and Shannon, 2005). The answers to the different questions in the interview-guides were condensed, summed up and compared across the regions. To validate the identified results, online meetings were arranged between researchers from the WHO CC MedUse and the interviewers in the regions. Three meetings were held with some of the interviewers from Archangelsk, Pskov and St. Petersburg to ensure that the analysis made by the WHO CC MedUse researchers was in line with the understanding of the regional interviewers. The process of conducting the interviews and the results were also discussed in these meetings. If researchers from WHO CC MedUse observed potential problems with data quality when doing the analyses (for example, noticing that many patients could name all types of antibiotics correctly, which was considered unlikely to happen in reality) then such issues were discussed at these online meetings. However, no corrections to the analysis were made as an outcome of those meetings.

### Ethical Considerations

An informed consent was obtained from every interviewee. Before the informed consent was obtained, the following information was shared with all interviewees: rationale, aim and objectives of the project, what will happen during the interview and how the interview will be used afterwards. The researchers from the WHO CC MedUse provided a template for written consent. Oral consent was accepted in the case of the interviewees feeling uncomfortable with written consent. Furthermore, in each region relevant ethical requirements for qualitative studies in the respective jurisdictions was followed. The Danish Data Protection Agency, effectively executed by the Faculty of Health and Medical Sciences, University of Copenhagen, granted permission for the WHO CC MedUse to store and process data (Ref. no:514-0389/19-3000).

## Results

This section presents the main results for patients with and without prescription of an AB across the five regions. For the total numbers of conducted interviews by regions, see Table 1.

**TABLE 1:** Interviews conducted per region.

	Patients *with* Rx	Patients *without* Rx	Total
Arkhangelsk	3	3	6
Karelia	8	6	14
Murmansk	5	5	10
Pskov	5	10	15
St. Petersburg	4	6	10
Total	25	30	55

### Patients With Prescriptions

As shown in Table 2, 25 patients with prescription agreed to participate in the study out of which six were men. The age span of these went from 18 to 56 years old and all had middle to high education levels.

**TABLE 2:** Overview of patients with prescription.

	Age	Gender	Place of work and education/profession
Arkhangelsk			
Patient 1	18	Female	College student
Patient 2	58	Female	Extracurricular education teacher
Patient 3	47	Female	Chief bank specialist
Karelia			
Patient 1	24	male	Higher education in construction and building
Patient 2	24	female	Higher pedagogical education
Patient 3	40	female	Seller
Patient 4	35	female	Higher economic education
Patient 5	36	male	Higher education, military officer
Patient 6	35	male	Mechanical engineer
Patient 7	55	male	Excavator operator
Patient 8	46	female	Junior childminder
Murmansk			
Patient 1	36	Male	Police officer
Patient 2	56	Female	Pensioner
Patient 3	39	Female	Elementary school teacher
Patient 4	48	Male	Military officer
Patient 5	22	Female	Student
Pskov			
Patient 1	20	Female	Student at the university
Patient 2	32	Female	Higher pedagogical education
Patient 3	23	Female	Vocational secondary education
Patient 4	42	Female	Higher education
Patient 5	40	Female	Higher legal education
Saint Petersburg			
Patient 1	34	Female	Higher economic education
Patient 2	32	Female	Higher economic education
Patient 3	28	Female	Computer scientist
Patient 4	52	N.D.	Clerk (vocational education)

ND, no data.

#### Common Symptoms Related to AB Use

Symptoms often reported by patients with prescriptions included runny nose, dry cough, fever, loss of voice and sore throat. Although the interviewees reported fever as a symptom, only a few of them gave the specific temperature. The duration of symptoms varied from 3 to 20 days*.* Often, patients waited from 5 to 7 days before going to the doctor, hence experiencing persisting symptoms. For example, one patient talked about having suffered from wet cough for several weeks. Initially she thought she had acute respiratory viral infection (ARVI), *“but when I experienced shortness of breath while climbing stairs, she thought that these symptoms were not like ARVI” (female, 52).*

#### Use of Leftovers, Checking Internet and the Influence of Friends and Family

Some patients said they would not use medicine from the home cabinet; one of the patients answered the question with “*No, it is better to consult a doctor to avoid complications. My mom is of the same opinion that it is better to go to the doctor. She is a doctor specialist and she knows better*” (*female, 18*). Others were of a different opinion “*I’ve always got a supply of ABs at home*” (*female, 58*)*.* This patient also stated frequent use of AB.

Few patients looked up symptoms online, but one specifically said he searched for the clinical guidelines*.* Others had used the Internet on previous occasions to find out how an AB works in the body and how to take them: “*I know that antibiotics do not always help and the organism can get used to it. I learned it from the Internet” (female, 39).* Other patients searched the Internet for information about their symptoms, however, the suggestions were inconclusive and for one even scary: “*I had some suspicions but did not know exactly what it was. As everyone else, I browsed the internet and there were a lot of scary things” (female, 22).*

Often patients discussed their symptoms with relatives including parents, children and spouse before seeking the doctor: *“Well, I talked with my daughter and she told me to better go to the doctor” (female, 56); or “I discussed them with my wife” (male, 48).* The recommendations that patients often received from their family members were to go to a doctor.

#### Particularities of Consultations With Doctors and Choice of AB

The reasons for going to the doctor were either to get a treatment for their persisting sickness, often when symptoms were unknown (or to get a confirmation of their own diagnosis), to get a sick leave or to get both treatment and a sick leave. Hence, visits to public sector doctors were more popular when the patient was in need of getting a note to justify absence from job or studies. During the consultations with doctors, patients said they were examined (throat examination and lung auscultation) and asked about symptoms. In some cases, tests (blood, urine or x-ray) complementing the doctor’s examinations were conducted at the time of the consultations.

Some patients said they did not receive a clear and specific diagnosis, some mentioned flu or ARVI. Others did not report any diagnosis, often as they did not remember it. Others reported specific diagnoses such as acute respiratory illness, acute laryngitis, follicular tonsillitis and maxillar sinusitis. The patients were almost never consulted in the choice of AB or treatment options, although they were given instructions on how to use AB: *“The doctor asked for the symptoms and listened to the lungs, prescribed treatment. He didn’t say the diagnosis, I didn’t ask” (female, 20).*

The most common AB chosen by doctors were amoxicillin and co-amoxiclav. Two patients specifically mentioned that they were in favor of the doctor’s decision to prescribe AB, thereby illustrating them reflecting over the doctor’s decision: “*Well, naturally it was a doctor who chose to prescribe me an AB, and I agreed” (female, 39).* The general impression was that, according to the patients, doctors spent more time explaining how to use the AB, than discussing why it was needed.

#### Encounter at the Pharmacy

Patients described the choice of pharmacy as based on convenience, either because they lived or worked close to the pharmacy or because of economic reasons (city budget pharmacy or having a discount card). Often patients reported that the pharmacists did not provide any instructions on how to use the AB or special considerations regarding the use of it.

#### Patients’ Knowledge About AB Treatments and Reactions Towards AMR Campaigns

When it comes to knowledge, patients often mentioned that ABs kill bacteria and some said that ABs do not work on viruses. For example, one patient said “*As people say, they kill germs and bacteria, but both good and bad” (female 58),* another patient said *“I think they just kill the infection. Probably, not only the infection, but they kill also good bacteria” (female, 47).* Some patients said that they did not know how ABs work in the body, while others described that ABs affect the body negatively. For example, that the body can get used to ABs and that it gets weaker: *I know that antibiotics do not always help and the organism can get used to it” (female, 39), “Once I heard that antibiotics destroy our own immunity” (female, 22).* A few patients described that ABs should be used only in severe cases, one patient additionally mentioned that it was acceptable only when prescribed by the doctor: *“When ABs are really necessary to a person and the doctor prescribes them” (male, 36).* Only one patient specifically referred to AB resistance because *“… when taking them in the case of a non-bacterial infection, it may result in antibiotic resistance later” (male, 24).*

The sources of knowledge mentioned were TV, advertising, internet, books and doctors. In general, patients had not noticed any AB campaigns. Few patients had seen a poster on ARVI announcing not to take antibiotics, *“but they did not affect me in any way” (female, 24)* or vaguely remembered some advertisements, but could not definitely say what those advertisements were about: *“Perhaps, I saw something similar on TV, but I didn’t remember anything” (female, 32).*

### Patients Without Prescription

As shown in Table 3, 30 patients without prescription agreed to participate in the study out of whom six were men. The age span went from 19 to 64 years old and all had middle to high education levels.

**TABLE 3:** Overview of Patients without Prescription

	Age	Gender	Place of work and education/profession
Arkhangelsk			
Patient 1	45	Female	Medical representative
Patient 2	63	Female	Technician meteorologist
Patient 3	32	Female	Higher economic
Karelia			
Patient 1	27	Female	Higher pedagogical education
Patient 2	24	Female	Vocational medical education
Patient 3	30	Male	higher legal education
Patient 4	53	Female	Vocational technical education
Patient 5	52	Female	Vocational secondary education
Patient 6	19	Female	2 nd year physical education
Murmansk			
Patient 1	35	Female	Military
Patient 2	39	Female	Military
Patient 3	48	Male	Security worker
Patient 4	40	Female	Sales assistant
Patient 5	40	Male	Military
Pskov			
Patient 1	34	Female	Higher pedagogical
Patient 2	47	Female	Vocational secondary
Patient 3	56	Female	Secondary technical
Patient 4	25	Female	Higher psychological
Patient 5	64	Female	Higher pedagogical
Patient 6	46	Female	Vocational medical education
Patient 7	63	Female	Secondary medical
Patient 8	41	Female	Higher psychological
Patient 9	36	Female	Higher economic
Patient 10	46	Female	Higher pedagogical
St. Petersburg			
Patient 1	24	N.D	Psychology student
Patient 2^a^	34	female	Primary school teacher
Patient 3^b^	41	female	Higher professional education
Patient 4	65	Male	Degree in engineer
Patient 5	47	female	Higher education (legal studies)
Patient 6	38	female	Higher medical education

a The medicine was for her grandmother, not for her

b She did have a prescription

### Common Reported Symptoms

Often patients without prescription experienced symptoms like severe sore throat, sneezing, cough, headache, fatigue and fever. The symptoms had lasted between 4 and 7 days before getting an AB: *‘I had my nose congested, running nose, constant sneezing, also fever and headache.’ (male, 48).* In many cases, patients had experienced the symptoms in an earlier illness episode and got a diagnosis (such as bronchitis, tonsillitis, maxillary sinusitis, pharyngitis), thus suspecting that the same was happening now.

#### Motivations to Avoid Consulting a Doctor

Most patients avoided going to the doctor because they did not want to take a sick leave or had no time: “*I did not have time for a doctor at all; I worked from 10 a.m. to 10-11 p.m.“(Female, 32).* Another mentioned that it is a waste of time, as the doctor seldom will prescribe AB or the patient knew for sure which AB they would prescribe: *“They prescribe the same things every time and I know what they will prescribe me” (female, 52).* Other reasons included that the condition was not serious enough to seek the doctor (*‘I thought the illness was not so serious to seek a doctor, and I felt myself satisfactory.’ (male, 48)*)*,* or that there was no need in as they themselves knew the diagnosis (“Y*ou only go to the doctor when experiencing specific symptoms for the first time: ‘Because this is not the first time this illness occurs’” (male, 40)*).

#### Use of Leftovers, Checking Internet and the Influence of Friends and Family

Approximately half of the patients checked the home medicine cabinet and found some ABs. One patient said: *“I checked and found two tablets of amoxicillin from a previous illness and started taking [it]” (female, 52).* Another found Ciprofloxacin and then started self-medicating with this for fever, cough and pain when swallowing: *“Of course [checking the home medicine cabinet], and I found a suitable AB. We bought it last time when our daughter fell ill” (female, 40).* However, the majority of participants responded that they checked their medicine cabinets, but did not find any AB or that the supplies were not sufficient. For example in one case, the cabinet was checked, one tablet of AB was found: *“I gave it to my husband. He felt better, so we decided that he needed to take a course of this AB” (female, 41).* Last, a patient used the online pharmacy store: *“Now there a very convenient thing—apteka. ru where you can order an AB. You will come and nobody will ask you for a prescription for an AB” (female, 45).*

A few patients looked up their symptoms online. Patients often discussed their symptoms with their relatives or partners before going to the pharmacy and were encouraged to take AB: ‘*Yes, I told to my friend and she said I needed an antibiotic.’ (female, 35).*

#### Choice of Pharmacy and Choice of ABs

Patients based their choice of pharmacy on whether the pharmacy would normally sell AB without a prescription: *“This is not the first time I have taken ABs, thus I already know where the pharmacies are strict, where they will immediately tell me: ‘Give me the prescription’ and where I can just come and buy an AB.“(female, 45).* However, there seemed to be different patterns of how encounters without AB in a pharmacy actually evolved. The most often reported pattern was when patients expected some resistance from pharmacists, and therefore prepared a mission of getting AB. For example, some patients used lies such that the prescription was forgotten at home. A few faked the prescriptions by writing a name of the drug on a paper that looked like a prescription. An interesting case was reported when one prescription was used more than once by different persons. The patient said: *“She [the mother] presented it, the drug was sold to her, but the prescription was not taken. Therefore, the next day the two of us [mother and daughter] went to the pharmacy and bought one more pack of the AB with the same prescription. They also demanded a prescription from us, we showed it, but nobody took it from us. And it was another seller, thus there were no problems with the purchase” (female, 17).* Another pattern was when patients knew exactly the kind and dosage of AB they needed and displayed absolute confidence when buying and AB at a pharmacy.

Most of the patients bought amoxicillin 500 mg. All patients remembered the exact name and dosage of the AB they bought. Other purchased ABs included amoxiclav, ceftriaxone, levofloxacin. The patients often choose the AB and the dosage based on what they saw on the Internet, according to rumours, or because s/he received a prescription for such AB in the past. In the majority of cases, the selling of antibiotics without prescription was unproblematic. Some patients said that “*Yes, you can buy it without a prescription in our region”*. Another patient said, *“I had been sick for a week and needed an antibiotic and that was all” (female, 53)*. Some patients reported using AB without prescription from 2 up to 5 times a year.

#### Patients’ Knowledge About AB Treatments, and Reactions Towards AMR Campaigns

Most patients talked about ABs killing infections, but only a few mentioned antibiotic resistance as a problem, and often they said that they did not have any sound knowledge about ABs. However, one patient mentioned that ABs cannot be used for viruses. Some patients answered that ABs should only be used when there are no other possible medicine to take, and others said that it should not be used when the body can recover by itself: *‘Well, when the organism copes itself and the illness is not serious’ (female, 39)*. Often, patients agreed that AB is a strong, quickly working drug, with side effects or addiction, to be used for serious conditions, when other remedies do not work: *‘When the other medicines do not help’ (male, 48).* Half of the patients said that optimally doctors should prescribe AB for a confirmed diagnosis, or at least someone with medical education should suggest ABs. Few patients knew about AB´s potential impact on the gut and half of the patients talked about allergy related to ABs.

Half of the patients said they did not see or did not remember any campaigns regarding use of ABs. One reported coming across a campaign: “*Well, yes, I read in the newspapers that it is advisable to consult a doctor in the case of a cold and not to self-medicate. And not start taking antibiotics on your own, as they can be unnecessary” (female, 52).* Two other patients described that they had noticed specific AB campaigns but had not paid any attention to them: *‘Well, there were some but I did not read carefully nor paid much attention.’ (male, 48)*. Two mentioned brochures they saw while waiting for doctors in polyclinics, four remembered advertisements (TV presumably), and one mentioned both. The knowledge the respondents said they gained from campaigns concerned prohibition to use AB unnecessary or without doctor’s prescription (which was considered complied with, as the prescription was gotten from a doctor for a similar situation that happened previously), and the fact that AB works on bacteria, but not viruses.

## Discussion

Self-medication did not seem an unusual practice across all five regions; however, in some cases, patients had to insist and persuade pharmacists to get an AB without prescription. Factors such as time spent going to the doctor, need of a sick leave and self-persuasion influenced the decisions whether or not to consult doctors for URTIs symptoms. Knowledge of AB and AMR was in general low; however, even some patients with high knowledge self-medicated at times. Family members and friends were often involved in decisions around how to handle symptoms of URTIs. Few patients had noticed AB campaigns, and very few said they had learnt something important from them.

### Consolidated Practice of Self-Medication

A consolidated practice of self-medication was identified. Taking into account the available literature from the Eastern Europe region, the results of this study are in line with previous ones (Kaae et al., 2016) highlighting the spread of self-medication practice. New results however appeared, in particular the scenarios described by patients using ABs without prescriptions and how they made pharmacists give them ABs anyway. Attention on how to support pharmacies and pharmacists to comply with current regulations appear to be of importance.

Further, some actions prior to consulting doctors or at the pharmacy, mainly identified by quantitative studies, were also explored in this study. For example, Stratchounski et al., in 2003 found that 83.6% of Russian families had antibiotics for systemic use in home medicine cabinets (Stratchounski et al., 2003). This study thus confirmed former identified practices such as: a) confirmation-seeking of symptoms by patients; b) how to respond appropriately to these by searching the internet and/or discussing it with friends and families; and c) checking/using ABs from the home cabinets. However, this study identified some new important elements in these practices. For example how patients can get both encouraged but also scared when searching the internet and their specific reflections about using leftover ABs. Hence, this study shows that these processes influence the patients’ further choice of action regarding use of AB, and therefore ought to be investigated more in-depth in the future.

### Low Awareness of AMR Related Threats

There was a large variation of knowledge about AB among the interviewees. The concept of good and bad microorganisms in the body seemed to be widespread among patients and some caution towards the use of AB was expressed. Patients however seldom addressed AMR. These results are in line with a recent Australian qualitative study focused on patients’ perception of AMR and how these perceptions influence the attitudes towards ABs. It showed that what actually becomes resistant is poorly understood. Moreover, both studies showed that although AMR might be perceived as a future big problem for the community, there is little appreciation of the individual impact to it (Bakhit et al., 2019). Our results also show that poor awareness of AMR and believing that antibiotics were effective for URTIs were more often reported among those participants using ABs without prescription. This insight is in line with the findings from Roope et al. in the quantitative study conducted in the UK through an online survey among the general public (Roope et al., 2015).

### Poor Impact of Scarce Campaigns

Although a few patients described specific messages given in the campaigns such as: the need to consult a doctor; ABs don´t kill viruses; not to start self-medicating with ABs, AB campaigns seemed to have very limited effect. The scarce impact of information campaigns or even the paradoxical consequence of information or awareness-raising campaigns has been previously documented (Bakhit et al., 2019; Röing et al., 2020). Bakhit et al. showed that information campaigns to reduce AMR might risk a paradoxical consequence of actually increasing public demand for antibiotics (Bakhit et al., 2019). The qualitative Swedish studies conducted by Röing et al. highlight the fact of designing better-suited and more inclusive public educational campaigns that put more emphasis on behaviour change (Röing et al., 2020).

Previous studies have shown a relatively weak negative association between individuals with sufficient health literacy and the likelihood of a recent history of AB use (Salm et al., 2018). Although we did not include health literacy in our study, we did find that sometimes a relatively goodknowledge of AB was not aligned with attitudes towards ABs. This might indicate that providing knowledge on AB use, and AMR alone does not suffice to contain the spread of AMR and that specific behaviour change actions are needed. This important learning outcome also should be considered when exactly designing better-suited future AB campaigns (Röing et al., 2020).

### Interpretation of Behaviours and Essential Differences Between Participants With and Without Prescription

Participants’ motivations to use AB for URTIs can be understood through the lens of four major phenomenological concepts: time, intentionality, perception and agency (SGaD, 2021). Time-related considerations seem to have influenced many participants to choose a shortcut and self-medicate. Confronted with similar symptoms that those experienced in the past, the need to use time to go to the doctor is perceived as a waste of time for those using ABs without prescription. It is also perceived as a waste of time to be sick, and the interviewees expressed the wish to go back to work as soon as possible and continue with their everyday duties and responsibilities and avoid loss of income due to illness. Time is also a determinant factor for those choosing to go to a doctor, especially for those wanting to get a sick leave, a paper documenting that they are actually sick and need to take days of work off, time off. Whether there is a correlation between the type of work or the socioeconomic status of the participant and the wish to safe time or take time off, our study cannot say, but time is definitively a category that showed up during many conversations with the interviewees. A further question could be, in the light of more appropriate knowledge about how AB works and the AMR threats, would these approaches to time change? Our data shows that even when an acceptable level of knowledge is present and appropriate, participants decided to self-medicate and thus use AB without any prove of their actual need. Time and its use seem to be a more decisive factor than a rational use of AB. As phenomenological concept, time and temporality are subjective, and so does illustrate our data. To change subjective perceptions of time and temporality seem to exceed any health promotion campaign targeted towards a more appropriate use of AB, as it is a category firmly embedded in our societal rhythms. Nevertheless, being aware of its importance might be useful to shape future awareness raising campaigns.

Participants’ behaviors are also driven by intentionality. Intentionality is intrinsically embedded in our mental and psychical realms. Many participants using ABs without prescription referred to a very clear pattern of intentionality when choosing a pharmacy to get AB, and it was clear to see that, beyond issues related to the prize of AB, knowing whether a given pharmacist will be more or less open to sell AB without prescription was a determinant factor for choosing a pharmacy. Participants’ intentionalities has given us a clear message: they will scan the landscape of available pharmacies until they find that specific one that will sell ABs without a prescription being presented, accepting hand written prescriptions, or not redeeming the prescription once the AB has been sold.

Intentionality can be however managed by strengthening and enforcing regulations *via* electronic prescription. This will substantially improve the control and thus the use of AB. It cannot however put an end to the use of leftovers or to getting AB from places other than pharmacies. Comparing patients with or without prescription, intentionality tend to differ. Our data points to stronger intentionality among those willing to get AB without prescription than among those consulting doctors, as they show a higher degree of accepting other persons’ intentionality, in this case that of doctors irrespective of how satisfied they were with how the consultation unfolded.

Our data shows that patients’ perceptions of the need for ABs are strongly linked with their behaviors, sometimes more than knowledge about AB. Phenomenology views perception as subjected to time and space and as highly influenced by the role of others. Perceiving a need for AB is thus subjective and changeable and it is time and space dependent. Participants expressed the importance of consulting others, but they also often expressed that they had already had the symptoms for at least 3 days or even longer. The waiting for recovery and the other’s view over their symptoms often influence their perception of being in need of ABs. The severity of symptoms is also subjective to perception and thus very personal. With the expectation of cases where AB was clinically justified, many of the cases described by the participants would land in a grey zone. The perception of severity of symptoms and thus in need of AB was not the determinant differential factor between patients with and without prescription, as it was the subjective perception of need. To tackle perceptions in healthcare is a considerable challenge that can often be solved by a point-of-care test rather than by conviction, knowledge or sensitization.

Patients’ actions are driven not by theoretical wondering of future consideration about AMR, but about practical concern, and this is what drives their sense of agency. In that sense, use of AB is the fruit of mere pragmatism. The phenomenology of agency sustain that, in order to cope, in order to take action, we do not need a very high order of consciousness, and that sometimes a very thin, pre-reflexive awareness suffice. We do see this pattern in the way the interviewees explained why they took action and went to the doctor or when directly to the pharmacy. An even more pragmatic approach can be intuit among those going directly to the pharmacy. It is perhaps unrealistic to expect deeper reflections for their rationale behind using AB without prescription. It would be perhaps more appropriate to expect a deeper reflection of their actions among healthcare professionals. A theme that will be discussed in follow up paper.

### Limitations and Strengths

This study have several limitations. Sampling of patients was not ideal, as it mostly was done by personal contacts or snowballing. The sample is relatively homogenous, sampling biases were observed towards middle aged, educated urban females. Previous research has shown this is the profile who often participate in surveys and interviews and is easiest to recruit and also more compliant with healthcare recommendations. Should the interviewers have been able to include a more diverse sample, we would have perhaps observe an even more widespread inapropirate use of antibiotics.

Besides, most interviewers were not experienced, neither in research interviewing nor in qualitative research as such (though most of the interviewers had participated in the above mentioned training session). In addition, despite quality actions taken, not all questions in the interview-guides were asked in all the interviews. Moreover, some expressions used by patients appeared unrealistic (for example, their use of very specific medical terminology or knowing the precise name and dosage of the antibiotic patients received). Furthermore, some interviewees did not fully understand all the questions, for example, the question on patient’s satisfaction with the process of getting the prescription and/or the purchase of ABs. A few examples of interviews where the interviewer did not seem to listen attentively to the interviewee were also observed, and in general only limited probing was applied (hence, interviewers in general asked questions but did not follow up on some answers that would benefit for deeper investigation). Because of these limitations, the interviews were less nuanced and detailed than expected.

The division of interviewers and analyzers, with the later not possessing sufficient knowledge about cultural aspects on ABs use in the different regions and the former being novices as qualitative researchers, might have led to misinterpretations, so that what was said during the interviews was interpreted with the analyzer´s limited knowledge of the cultural context. Although outsiders can see patterns not detected by those inside a context, some nuances have presumably been lost.

As to the strengths of the study, the results are in many ways comparable to former studies pointing at a culture of AB use as the answer to many symptoms common for many contexts (Stratchounski et al., 2003; Hsieh and Shannon, 2005; Roope et al., 2015; Salm et al., 2018; Bakhit et al., 2019; Röing et al., 2020; SGaD, 2021). The interviews revealed many relevant and new aspects of the use of AB in Northwest Russia, notwithstanding the described limitations. The results reveal how different aspects take place in the daily life of patients handling of ABs in Northwest Russia. The extent to which these practices take place, cannot be estimated based on this study, though. To this end, quantitative methods have to be employed. Further, as is the case with qualitative research, the results cannot and should not be generalized, but rather should be subject to a data quality assessment concerning trustworthiness and authenticity (Lincoln and Guba, 1986; Amin et al., 2020). As to the methodological limitations, some actions were taken to counteract them: including peducation of interviewers, feed-back given after a few interviews, and validation meetings between researchers and local regions (as described in the methods section).

When it comes to the authenticity criteria, the study also scores relatively high, because meanings have been developed (ontological authenticity) and there is a learning potential from the study (educative and catalytic authenticity). This study has thus highlighted the types of parameters, which would be relevant for inclusion in quantitative studies. The study can likewise be used to guide future initiatives to tackle AMR, based on the confirmation and an increased insight into what can be estimated as typical AB knowledge, attitudes and practices.

### Recommendations for Research, Health Policy and Health Promotion

When looking at the implications of this study for health promotion, it seems as campaigns have failed to instill enough awareness about AB use and AMR, at least for those persons using ABs. Future campaigns need to address the needs of the consumer in such a way as to engage them in the public health threat of AMR. The future approach calls for co-creation with public participation on how to best frame educational efforts that is relevant to the public. Our study showed that the interviewees have some, albeit little, knowledge that AMR can result from AB use in the “individual’s body”. However, there is little or no understanding that AB use in the individual can increase AMR in the “society’s body”. As Charani et al. (2021) suggest the design of campaigns for restrictive AB use can emulate the climate campaigns’ success of creating space for open dialogue and discussion among citizens and experts (researchers).

This study also showed that the choices to use AB were based on pragmatic reasoning of the interviewees who chose not to consult a doctor, i.e. that time is limited and therefore AB are sought directly from pharmacies as a form of self-care. This pragmatic reasoning needs to be tackled in any policy program intended to improve the public perceptions and behaviors surrounding AB. One such program could entail increasing the availability of teleconsultations with doctors.

On the supply side, our study points out that technical solutions can be implemented to stem inappropriate AB use, including electronic prescribing and point-of-care (POC) tests. Implementing these solutions may not be feasible in all settings, including community pharmacies. Research needs to be done of the cost-effectiveness of POC tests and in which locations testing could be done in the health care system. The results in this study show that many respondents based their decisions on AB use on the symptoms experienced and relate these to symptoms in prior illness episodes. Therefore, if POC tests are provided, patients need to understand what the implications of this biomarker mean in relation to the clinical symptoms they experience. Furthermore, the need for structural changes, e.g. prescribing systems is as aspect that has been highlighted as supreme importance (Charani et al., 2021).

Lastly, our study showed that, according to the interviewees no health care setting or professionals provided information to patients regarding prudent use of AB and the risk of AMR to the individual patient. Any health policy aimed at the user of AB needs also to incorporate the role of health care professionals in all settings - general practice, hospital and community pharmacies. A multi-lateral approach is also needed to ensure that all professionals have a shared role in appropriate use of ABs. In other words, no profession or setting can alone tackle the decision to use AB and education about AMR. This need for health care professional effort calls for education efforts both pre- and post-graduation, within professions and interprofessionally.

## Conclusion

Several features of patients’ knowledge, attitudes and regarding AB use in daily life in Arkhangelsk Region, Karelia Republic, Murmansk Region, Pskov Region and St. Petersburg City have been documented through carrying out and analyzing 55 interviews. Many of the identified patterns can be argued to be involved in increasing the risk of AMR. These include patients’ reliance on own judgements and self-medication, that may lean on previous illness and therapy history, information from the Internet or family and friends. Other patterns identified were assumed lack of time to see the doctor, relatively easy access to ABs without prescription at pharmacies or leftovers in medication cabinets at home, not enough knowledge about proper use of ABs. These findings can serve as a basis to build future initiatives to pursue proper use of ABs and hence tackle AMR in the regions.

## Data Availability

The raw data supporting the conclusions of this article will be made available by the authors, without undue reservation.
